# Regional Hurst Exponent Reflects Impulsivity-Related Alterations in Fronto-Hippocampal Pathways Within the Waiting Impulsivity Network

**DOI:** 10.3389/fphys.2020.00827

**Published:** 2020-07-10

**Authors:** Susanne Neufang, Atae Akhrif

**Affiliations:** ^1^Department of Psychiatry and Psychotherapy, Medical Faculty Heinrich-Heine University, Düsseldorf, Germany; ^2^Comparative Psychology, Institute of Experimental Psychology, Heinrich-Heine University, Düsseldorf, Germany; ^3^Center of Mental Health, Department of Child and Adolescent Psychiatry, University of Würzburg, Würzburg, Germany

**Keywords:** Hurst exponent, fMRI, neural network connectivity, impulsivity, fronto-hippocampal pathways

## Abstract

In general, the Hurst exponent. is used as a measure of long-term memory of time series. In previous neuroimaging studies, *H* has been introduced as one important parameter to define resting-state networks, reflecting upon global scale-free properties emerging from a network. *H* has been examined in the waiting impulsivity (WI) network in an earlier study. We found that alterations of *H* in the anterior cingulate cortex (*H*^*ACC*^) and the nucleus accumbens (*H*^*NAcc*^) were lower in high impulsive (highIMP) compared to low impulsive (lowIMP) participants. Following up on those findings, we addressed the relation between altered fractality in *H*^*ACC*^ and *H*^*NAcc*^ and brain activation and neural network connectivity. To do so, brain activation maps were calculated, and network connectivity was determined using the Dynamic Causal Modeling (DCM) approach. Finally, 1–*H* scores were determined to quantify the alterations of *H*. This way, the focus of the analyses was placed on the potential effects of alterations of *H* on neural network activation and connectivity. Correlation analyses between the alterations of *H*^*ACC*^/*H*^*NAcc*^ and activation maps and DCM estimates were performed. We found that the alterations of *H* predominantly correlated with fronto-hippocampal pathways and correlations were significant only in highIMP subjects. For example, alterations of *H*^*ACC*^ was associated with a decrease in neural activation in the right HC in combination with increased ACC-hippocampal connectivity. Alteration in*H*^*NAcc*^, in return, was related to an increase in bilateral prefrontal activation in combination with increased fronto-hippocampal connectivity. The findings, that the WI network was related to *H* alteration in highIMP subjects indicated that impulse control was not reduced *per se* but lacked consistency. Additionally, *H* has been used to describe long-term memory processes before, e.g., in capital markets, energy future prices, and human memory. Thus, current findings supported the relation of *H* toward memory processing even when further prominent cognitive functions were involved.

## Introduction

The Hurst exponent *H* is used as a measure of long-term memory of time series (Eke et al., 2000). In previous neuroimaging studies, *H* has been introduced as one important parameter to define resting-state networks (Taylor et al., 2012), reflecting upon global scale-free dynamics emerging from a network (Mukli et al., 2018). As real-world time series in general and neuroimaging data in particular, often do not fulfill the criteria of self-similarity or other fractal properties, fractal methods have been further extended. For example, long-range dependence and self-similarity have been shown to be strongly interrelated (e.g., Pipiras and Taqqu, 2017), thus, the application of fractal approaches on time series showing long-range dependence has been encouraged (Abry et al., 2013). Furthermore, the irregularity of a signal plays a crucial role, and that “this irregularity contains meaningful information” (Abry et al., 2013, pp. 19–20).

Among other cognitive functions, *H* has been linked to human memory processing mathematically (Namazi, 2018) as well as in numerous neuroscientific studies. For example, Wink et al. (2006) showed in a resting-state fMRI study, that bilaterally in the hippocampus (HC) *H* increased with age (Wink et al., 2006) in healthy subjects. In addition, *H* seemed to be a sensitive parameter to detect changes in HC processing in patients with and without memory disturbances, e.g., reduced hippocampal *H* in patients with mild cognitive impairment (Long et al., 2018), autism spectrum disorder (Dona et al., 2017a), and mild traumatic brain injury (Dona et al., 2017b). Furthermore, (multi)fractal analysis of fMRI has been used to “disentangle functional components from artifactual ones, in a robust and significant manner.” (Ciuciu et al., 2012) and to differentiate between healthy neural network from impaired ones at the example of the waiting impulsivity (WI) network in an earlier publication (Akhrif et al., 2018). WI is defined as the ability to inhibit a response in order to earn a reward (Voon et al., 2014). The WI network includes the ventromedial and dorsolateral prefrontal cortex (dlPFC) representing motor or response inhibition (Mechelmans et al., 2017), the reward perception-related nucleus accumbens (NAcc), the anterior cingulate cortex (ACC) for the cognitive evaluation of the reward and HC and amygdala (AMY) responsible for reward-based learning (Dalley et al., 2011). In an earlier study, we found that, *H* in the ACC and NAcc was reduced in high impulsive (highIMP) compared to low impulsive (lowIMP) participants (Akhrif et al., 2018). Very recent studies showed, that functional connectivity between the ACC and NAcc in the WI network varied in function of monetary reward (high reward, strong connectivity, and vice versa, Mechelmans et al., 2017) and that especially *H* in the ACC was associated with impulsivity (Gentili et al., 2020).

Following up on the earlier findings, we addressed the relation between the impulsivity-related *H* reductions in the NAcc and the ACC and brain activation and neural network connectivity of the entire WI network in this study. To do so, brain activation maps were generated, and network connectivity was determined using the Dynamic Causal Modeling (DCM) approach. DCM quantifies the influence region A has on a second region B; thus, it reveals the causal structure of a network. In a final step, *H* scores as described earlier were transferred into deviations from 1*H*. *H* valued close to 1 in the fMRI signal has been associated with highly complex and well attuned dynamics in neural networks (Lipsitz and Goldberger, 1992; Goldberger et al., 2002). In the earlier publication we showed that *H* values for highIMP subjects (i.e., impaired network functioning) were significantly lower than in lowIMP subjects. Therefore, the question to tackle in the current analyses was, how the deviation from 1 was related to brain activation and effective network connectivity. To address the relation between altered fractality and the network, correlation analyses were performed using *H* deviations and brain activation maps as well as DCM estimates. This way, the focus of the analyses was placed on the potential effects of alterations of *H* on neural network activation and connectivity.

## Materials and Methods

### Subjects

In this pilot study, we examined 103 male students, aged between 19 and 28 years (24.0 ± 2.6 years). Volunteers were recruited at the University of Wuerzburg, Germany, and screened for impulsivity using the Wender-Reimherr-Interview and Attention-Deficit/Hyperactivity Disorder checklist (subscales “impulsivity” and “hyperactivity and impulse control”; Rösler et al., 2008; for details see Neufang et al., 2016). The study was conducted in accordance with the Declaration of Helsinki in its latest version from 2008 and was approved by the ethics committee of the Faculty of Medicine, University of Wuerzburg. Their written informed consent was obtained from all volunteers.

### Experimental Paradigm

As cognitive task, the human version of the five choice serial reaction time task (5-CSRTT; animal version 5-CSRTT: Bari et al., 2008; human version, 4-CSRTT: Voon et al., 2014) was used. A trial started with a short presentation of 4 boxes, followed by a target in terms of a green dot, located in one of the four boxes. Correct and quick responses were reward by two amounts of money (10 Cent, 1 Euro). Premature responses were defined as reactions before target onset (for a representative trial see Supplementary Figure S1). The task consisted of one block outside the scanner (2.5 min) and five blocks within the MR scanner (14 min) with each block consisting of 20 trials. Total task duration was 16.5 min (for further detail Neufang et al., 2016).

Behavioral testing started with a first baseline block outside the scanner, conducted to determine the individual mean reaction time window (rt, M_rt_ ± 2 SD). The individual rt windows were used for reward definition in all consecutive blocks, which were performed in the MR scanner: one Euro if the subject responded correct and faster than the individual rt window, 10 cent if the subjects’ responses were within the same. Incorrect answers were neither rewarded nor punished.

### Data Acquisition

MRI scanning was performed using a 3 Tesla TIM Trio Scanner (Siemens, Erlangen, Germany). Functional MRI included a T2^∗^-weighted gradient echo-planar imaging sequence with the following parameters: repetition time (TR) = 2000 ms, echo time (TE) = 30 ms, 36 slices of 3 mm thickness, field of view (FoV) = 192 mm, flip angle = 90, and number of volumes = 425.

### Data Processing and Time Series Extraction

**fMRI-data processing** was performed using the Statistical Parametric Mapping Software Package (SPM12, Wellcome Department of Imaging Neuroscience, London, United Kingdom, Wellcome Trust Center for Neuroimaging; http://www.fil.ion.ucl.ac.uk/spm/). Data preprocessing in the native space included temporal and spatial alignment (i.e., slice time correction, realignment, and unwarping). Images were then spatially normalized into a standard stereotactic space (Montreal Neurological Institute), resampled to an isotropic voxel size of 2 mm × 2 mm × 2 mm, and spatially smoothed with a Gaussian kernel of 8 mm full width at half maximum. Pre-processing did not include high pass filtering or global mean correction. Model specification on single subject level included the experimental condition “response inhibition” and “reward” with *response inhibition* being related to target processing and reward was determined in terms of “win trials – loss trials.” In addition to the experimental conditions, “error trials” and “realignment parameters” (i.e., six regressors containing movement in three spatial and three rotational axes) were specified as nuisance regressors to reduce error variance and correct for movement artifacts. Condition-specific onset times were extracted from experimental log-files with onsets of the target trials defined at the moment, the target picture appeared, and onset times of reward trials locked to the time points when the reward feedback picture appeared on the screen. The onsets of error trials were defined as the target onsets of incorrect trials.

#### Time Series Extraction

Exact coordinates of ROIs were defined based on the significantly activated brain regions of “response inhibition”- and “reward” processing resulting from one sample *t*-tests. The local maxima of each significantly activated regions were identified and coordinates were then used as the center of a 10 mm spheric ROI using MarsBar (Brett et al., 2002). ROIs were built and used for the extraction of the time series for each subject. Time series extraction was performed using the routine as suggested by Brett et al. (2002)^1^ from preprocessed fMRI data (i.e., smoothed files resulting from the pre-processing procedure; Brett et al., 2002). Finally, linear trend removal was performed between the first and the last data point of the extracted time series [using the matlab routine detrend (*y*)] (Bai et al., 2008; Zhang et al., 2008; Fox et al., 2009; Qiu et al., 2011). Linear detrending is a necessary pre-processing step, as fMRI time series have slowly varying trends, that should be removed before performing spectral analyses (Tanabe et al., 2002).

2.5 adaptive fractal analysis- AFAMultiple investigations showed that long memory is an attribute, a property of functional networks and *H* is the mathematical expression, used to quantify it. Long memory processes belong to a wider range of processes, all expressing a power law spectrum (Eke et al., 2000). Their power spectral density function

(1) $$S\,x\,\left(-\right)\,S\,x\,\left(f\right)\approx C\,{\left|f\right|}^{-\beta }\;\text{where}-1<\beta <3$$

[β^ACC^: *M* = 0.86 ± 0.24, *T*(103,1) = 91.01, *p* = 0.000; β^NAcc^: *M* = 0.81 ± 0.26, *T*(103,1) = 85.04, *p* = 0.000] and with the approximation improving as *f* approaches zero. Whereas fMRI signals reflect information stemming from different cognitive and physiological processes [e.g., respiratory-frequency: 0.1–0.5 Hz; cardiac-frequency range: 0.6–1.2 Hz (Cordes et al., 2001); cognition-related low-frequency band: 0.045–0.087 Hz (Yang et al., 2018)], neural activity as providing the basis of functional connectivity in particular (Biswal et al., 1995) is carried in the low frequency components of the fMRI signals (<0.1 Hz; Achard et al., 2006; Ginestet and Simmons, 2011). Such processes describe scale-free, or scale-invariant time dynamics such as temporal brain activities. Scale invariance is associated with long range correlation in time. This is the condition to check for first, to assume scale invariance. To compute the β exponent, however, different definitions and methods might be used. For β values interpretation (Eke et al., 2002) as well as the class of processes related to them see Akhrif et al. (2018).

In this study, AFA was chosen for the determination of *H*, a factor that reflects in a power law manner the relationship, that is intrinsic to fractal processes, between the variance of fluctuation computed around, in our case, a second order polynomial trend *v* (*i*) fitted to time series within each segment *w*, and its size:

(2) $$\begin{array}{c} F\,\left(w\right)={\left[\frac{1}{N}\,\sum_{i=1}^{N}{\left(u\,\left(i\right)-v\,\left(i\right)\right)}^{2}\right]}^{1/2}∼w^{H}\\ N:l\,e\,n\,g\,t\,h\,o\,f\,t\,h\,e\,t\,i\,m\,e\,s\,e\,r\,i\,e\,s\\ w=2\,n+1,n=5,6,\ldots ,13 \end{array}$$

*H* was defined as the slope of the log-log diffusion plot *log*_2_(*F*(*w*)) as a function of *log*_2_(*w*) (for further details see Akhrif et al., 2018).

According to the dichotomous fGn/fBm model of Mandelbrot and Van Ness (1968) as introduced in the fractal time series analysis field by Eke et al. (2000, 2002), signal classification was performed (Mandelbrot and Van Ness, 1968; Eke et al., 2000, 2002). One of three methods of signal classification was detrended fluctuation analysis (DFA) of Peng et al. (1994). Since AFA results (*H*), with AFA being strongly related to DFA, were significantly smaller than 1 in both regions [*H*^ACC^: *M* = 0.93 ± 0.12, *T*(103,1) = 5.95, *p* = 0.000; *H*^NAcc^: *M* = 0.91 ± 0.13, *T*(103,1) = 7.22, *p* = 0.000]s, signals were classified fGn, and *H* = *H*_*fGn*_.

### Brain Activation

On single subject level, two contrasts of interest were calculated, “response inhibition” to isolate target-induced brain activation, and “reward” in terms of “win-loss” to identify brain activation associated with the receipt of monetary reward. All brain analyses were performed in a region of interest (ROI) based approach, using atlases within the Wake Forest University PickAtlas toolbox,^2^ and covering the WI network regions: bilateral superior frontal gyrus, MFG, orbital, triangular, and opercular parts of inferior frontal gyrus (IFG), ACC, HC, and AMY, NAcc, and medial fronto-orbital gyrus. Resulting contrast images entered statistical group analysis.

### Neural Network Connectivity (DCM)

For DCM analysis, DCM 12 was used as implemented in the SPM12 software. The current network included eight regions, resulting from significantly activated WI network regions (see Table 1). Endogenous connectivity and the condition-specific (i.e., response inhibition, reward) modulation of connectivity (modulatory inputs) were addressed. Subject-specific coordinates of the global maxima of activated clusters from brain activation results were used as centers for ROIs. Volume of interest spheres with a radius of 5 mm were built around the averaged coordinates in the NAcc and the AMY, and with a radius of 8 mm in all cortical regions. Different sphere sizes were chosen due to the regional volume size of the structures. Regional time series were extracted for all network regions.

**TABLE 1:** Global maxima of WI-associated brain regions.

Condition	Brain region			*Z*	Localization
	*x*	*y*	*z*		
Response inhibition	24	−28	−6	21.3	Right HC
	−22	−28	−6	17.7	Left HC
	−44	6	28	21.7	Left MFG
	40	8	34	19.7	Right MFG
	6	30	28	18.6	Right ACC
	8	12	−10	14.6	NAcc
Reward	−10	8	−14	14.7	NAcc
	−22	0	−12	5.5	Left amygdala
	0	48	−12	6.5	vmPFC

*ACC, anterior cingulate cortex; HC, hippocampus; MFG, middle frontal gyrus; NAcc, nucleus accumbens; vmPFC, ventro-medial prefrontal cortex, *p* < 0.05, and FWE-corrected on voxel-level.*

Based on introduced findings, ten model families were constructed with 4 families varying connectivity within response inhibition-related network, 4 reward-associated families and 4 families across both conditions. Across all families and models, endogenous connectivity was specified for all connections, conditions-specific modulation, however, was varied as follows.

Inhibition-related families were families one to four. In family one (HC bottom-up), it was assumed that the HC influences top-down regions such as right and left MFG (family 1, model 1), the ACC (family 1, model 2), and both, MFG and ACC (family 1, model 3). Families two to four varied top-down connections bilateral from the MFG on the HC (family 2), from the ACC on HC (family 3), and MFG and ACC on the HC (family 4).

Families five to eight defined the interplay between the NAcc, AMY, and the vmPFC. Therefore, family five determined the bottom-up signaling of the NAcc to the AMY (family 5, model 1), to the vmPFC (family 5, model 2), and both, the AMY and vmPFC (family 5, model 3). In analogy, in family 6 the AMY was defined as bottom-up structure and models varied between the targeted region NAcc (family 6, model 1), vmPFC (family 6, model 2), and both, NAcc and vmPFC (family 6, model 3). In family 7, both, NAcc and AMY were defined as bottom-up structure signaling to the vmPFC (family 7, model 1). In family eight, reward-associated top-down was defined on the NAcc (family 8, model 1), on the AMY (family 8, model 2), and on both, NAcc and AMY (family 8, model 3).

Across conditions, connections between reward-associated structures NAcc and AMY and inhibition-related MFG and ACC were defined in terms of family 9: NAcc – bottom-up signaling to the MFG (family 9, model 1), the MFG and the ACC (family 9, model 2), the MFG and the HC (family 9, model 3) as well as the MFG, the HC and the ACC (family 9, model 4). Family 10 defined HC and NAcc combined bottom-up signaling. Families 11 to 13 varied MFG and ACC top-down modulation of NAcc and AMY (for all models see Supplementary Table S1).

The families covering 28 models were compared applying random-effects Bayesian model selection (Stephan and Friston, 2010; Stephan et al., 2010) within a pre-specified Occam’s window (*p* < 0.05). Individual parameter estimates of the model with highest evidence were then assessed by means of random-effects Bayesian model averaging (Penny et al., 2010) across the models of the winning family. The Bayesian model averaging parameter estimates were then entered into summary statistics at the group level. The significance of each parameter was assessed by a one-sample *t*-test To test condition-specific modulation of connectivity for significance, repeated measure ANOVA models were defined with the within-subject factor *connectivity type* (endogenous connectivity vs. condition-specific modulatory input). Threshold for statistical significance was *p* < 0.05, FDR-corrected for multiple comparisons (Benjamini and Hochberg, 1995).

### Statistical Analysis: Brain Activation and AFA

To address the relation between *H*_*fGn*_ deviation and network function, 1−*H*_*fGn*_ scores were calculated. On group level, two sample *t*-tests were defined with the group factor impulsive phenotype (highIMP vs. lowIMP) including the covariates $H_{f\,G\,n}^{A\,C\,C}$ and $H_{f\,G\,n}^{N\,A\,c\,c}$ alterations and determined as interacting with the group factor. Contrast of interest were (i) the correlation between alterations in *H*_*fGn*_ and brain activation across all subjects (e.g., response inhibition ^∗^ $H_{f\,G\,n}^{A\,C\,C}$ alterations) as well as (ii) group-specific correlations (e.g., response inhibition^∗^ $H_{f\,G\,n}^{A\,C\,C}$ alterations: highIMP vs. lowIMP). The between-subject factor *impulsivity* classified subjects based on behavioral performance [i.e., a number of premature responses ≥ 3 in the 5-CSRTT as highIMP (*n* = 38) subjects and subjects with number of premature responses < 3 as lowIMP (*n* = 65) subjects]. Threshold of significance was p_*FWE*_ < 0.05 on voxel level.

## Results

### fMRI Analysis

One sample *t*-tests of *response inhibition* and *reward* revealed a significantly activated WI network including the regions right/left HC, right/left MFG, ACC, right/left NAcc, left AMY, and the vmPFC (Table 1).

### Dynamic Causal Modeling Estimates

Model comparison favored the reward-related NAcc + Amy bottom-up model 1 of family 7, with a family exceedance probability of xp = 0.8353 and a model exceedance probability of xp = 0.9954. In the winning model, endogenous connectivity included all connections, and reward-related modulatory input bidirectional connectivity between the NAcc and the AMY and going to the vmPFC (i.e., NAcc→l_AMY, NAcc→vmPFC, l_AMY→NAcc, and l_AMY→vmPFC). The one-sample *t*-test, identifying connections of significant connectivity strength revealed that almost all connections were passed the threshold of significance except for connectivity from the rHC→l_AMY (*T* = 1.3, *p* = 0.182, n. s.), lMFG→rHC (*T* = 1.1, *p* = 0.275, n. s.), r_MFG→vmPFC (*T* = 0.8, *p* = 0.458, n.s.), ACC→r_HC (*T* = 1.1, *p* = 0.267, n.s.), and vmPFC→r_MFG (*T* = 0.6, *p* = 0.582, n.s.). Repeated-measures ANOVA revealed significant modulation for all four connections (NAcc→l_AMY: F_con__nectivity type_ = 37.5, *p* = 0.000; NAcc→vmPFC: F_connectivity type_ = 114.7, *p* = 0.000; l_AMY→NAcc: F_connectivity type_ = 40.5, *p* = 0.000; and l_AMY→vmPFC: F_connectivity type_ = 72.6, *p* = 0.000).

### Alterations in $H_{\mathrm{fGn}}^{\mathrm{ACC}}$ and Network Function

**FIGURE 1:**
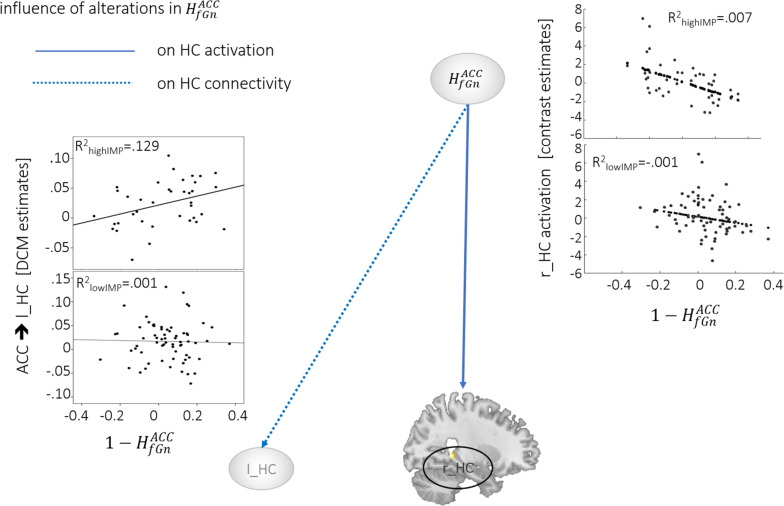
Neural activation and connectivity associated with $1-H_{f\,G\,n}^{A\,C\,C}$. Dotted lines indicate correlations with brain activation, arrows represent correlations with connectivity. Scatterplots show correlations, specifically for highIMP, and lowIMP individuals.

During *response inhibition* brain activation in the r_HC negatively correlated with alterations in $H_{f\,G\,n}^{A\,c\,c}$ across all subjects. The effect, however, seemed to be driven by highIMP subjects as demonstrated in the scatterplot in Figure 1. In addition, highIMP-specific positive correlations between connectivity emerging from the ACC and heading toward the l_HC (ACC→l_HC) and alterations in $H_{f\,G\,n}^{A\,c\,c}$ were revealed. Furthermore, in lowIMP subjects $H_{f\,G\,n}^{A\,c\,c}$ alterations correlated negatively with left-hemispheric fronto-hippocampal connectivity, i.e., l_HC bottom-up signaling to the l_MFG and frontal top-down control of the l_HC by the l_MFG (l_HC→l_MFG, l_MFG→l_HC; for all results see Tables 2, 3 and Figure 1).

**TABLE 2:** Significant correlations between alterations in *H*_*fGn*_ and brain activation.

Condition	Contrast	Brain region			*Z*	Localization
		*x*	*y*	*z*		
Response inhibition ^∗^ $1-H_{f\,G\,n}^{A\,C\,C}$	(lowIMP + highIMP)^(–)^	26	−42	0	4.1, *p* = 0.054	Right HC
Reward ^∗^ $1-H_{f\,G\,n}^{N\,A\,C\,C}$	highIMP > lowIMP	−42	12	42	4.8, *p* = 0.004	Left MFG
		40	20	44	4.6, *p* = 0.006	Right MFG
		50	34	−2	4.4, *p* = 0.018	Right IFG
		−44	18	8	4.3, *p* = 0.022	Left IFG

*^(–)^, negative correlation; HC, hippocampus; MFG, middle frontal gyrus; IFG, inferior frontal gyrus; p. triangularis, *p* < 0.05, and FWE-corrected on voxel-level.*

**TABLE 3:** Significant correlations between alterations of *H*_*fGn*_ and network connectivity.

	Connection	*R*_lowIMP_	*R*_highIMP_	*Z*
$1-H_{f\,G\,n}^{A\,C\,C}$	l_HC→l_MFG	−0.32*, *p* = 0.01	0.23, *p* = 0.17	2.6*, *p* = 0.01
	l_MFG→l_HC	−0.30*, *p* = 0.02	0.21, *p* = 0.21	2.4*, *p* = 0.01
	ACC→l_HC	−0.03, *p* = 0.84	0.36*, *p* = 0.03	1.9*, *p* = 0.03
$1-H_{f\,G\,n}^{N\,A\,C\,C}$	l_MFG→r_HC	−0.09, *p* = 0.49	0.35*, *p* = 0.03	2.1*, *p* = 0.02

*l_HC, left hippocampus; r_HC, right hippocampus; l_MFG, left middle frontal gyrus; ACC, anterior cingulate cortex; ^∗^*p* < 0.05; FDR- corrected for 64 comparisons (8 network regions, 8 × 8 connectivity matrix).*

### Alterations in $H_{\mathrm{fGn}}^{\mathrm{NAcc}}$ and Network Function

**FIGURE 2:**
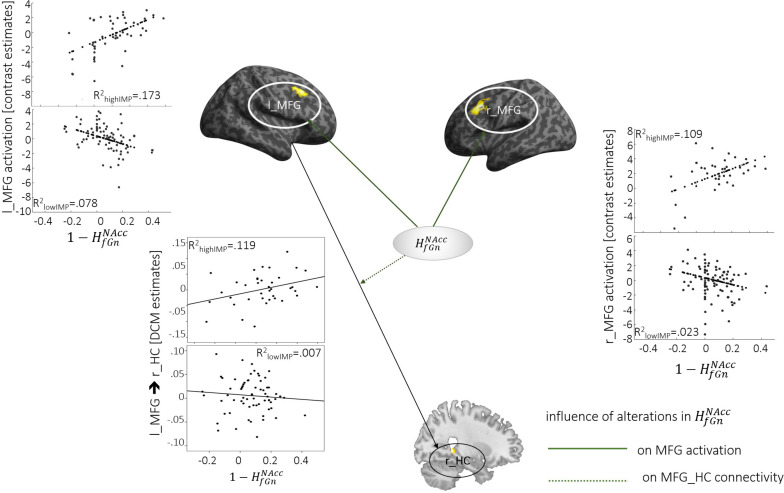
Neural activation and connectivity associated with $1-H_{f\,G\,n}^{N\,A\,C\,C}$. Dotted lines indicate correlations with brain activation, arrows represent correlations with connectivity. Scatterplots show correlations, specifically for highIMP, and lowIMP individuals.

*Reward*-specific activation bilaterally in the dlPFC (MFG and IFG pars triangularis) correlated positively with $H_{f\,G\,n}^{N\,A\,c\,c}$ alterations. This correlation was stronger in highIMP compared to lowIMP (see Tables 2, 3 and Figure 2). In addition, alterations in $H_{f\,G\,n}^{N\,A\,c\,c}$ correlated in highIMP but not in lowIMP subjects, with top-down control of the r_HC by the l_MFG (l_MFG→r_HC).

## Discussion

In this study, we investigated the relation between impulsivity-associated deviations of *H*_*fGn*_ in the ACC and the NAcc and the function of the underlying network. In an earlier publication, *H*_*fGn*_ in both regions have been found to be reduced in highIMP subjects. Current analyses revealed that deviations in the $H_{f\,G\,n}^{A\,C\,C}$ were predominantly associated with response inhibition processing, namely right hippocampal brain activation and, specifically in highIMP subjects, connectivity from the ACC on the left HC. Likewise, deviations in $H_{f\,G\,n}^{N\,A\,c\,c}$ were associated with broad reward-associated activation clusters in the dlPFC as well as connectivity from the left MFG on the right HC. Findings in reward processing were only significant in highIMP subjects. Thus, across all analyses, *H*_*fGn*_alterations seemed to be related to HC functioning, hinting toward a *H*_*fGn*_ -specific relation to HC processing.

In the WI network as proposed by Dalley et al. (2011) the HC has been assumed to directly interact with the ACC in terms of both structures top-down controlling the NAcc. Empirical evidence in humans, however, is scarce (Morris et al., 2016; Neufang et al., 2016; Mechelmans et al., 2017). Recent studies showed, that impulsivity was strongly linked to a hypothalamo-hippocampal pathway including the HC (Gruber and McDonald, 2012), and the NAcc (Barlow et al., 2018; Noble et al., 2019) with a maladaptation of HC-NAcc pathway contributing to the development of impulsivity and impulsivity-associated psychiatric disorders such as addiction (Everitt and Robbins, 2005). For example, in mice WI behavior potentiated hippocampal neurogenesis the way that during reward seeking behavior in the 5-CSRTT, precursor cells were produced in the dentate gyrus of the HC (Oliveros et al., 2017; Peyton et al., 2019). Such impulsivity-induced neurogenesis has been discussed as reflecting both reward-driven highIMP responding and (the known HC-associated) heightened learning performance (Peyton et al., 2019). Thus, it seemed, as if HC function plays a crucial role within the impulsivity network. Combining both assumptions, (i) the HC plays a crucial role within the network and (ii) fractal parameter such as *H* disentangling functional components from artifactual ones, the current strong relation between *H*_*fGn*_ and HC processing seemed plausible. An alternative explanation might be their common involvement in long-term memory. *H* has been used to describe long-term memory processes before, e.g., in capital markets (Di Matteo et al., 2005; Granero et al., 2008), energy future prices (Serletis and Rosenberg, 2007), and human (motor) memory (Chen et al., 1997; Namazi, 2018). The 5-CSRTT, in return, involves a high learning and memory load as the training and test protocol for animals covers numerous training sessions over weeks (“approximately 30–40 daily sessions,” Bari et al., 2008). Thus, in our analyses, the *H*_*fGn*_ exponent proved its strong relation to learning and memory processing even when further prominent cognitive functions such as reward processing were involved.

In addition to HC, altered *H*_*fGn*_ was associated with frontal activation and connectivity. The MFG and the IFG are core regions within impulsivity and WI (Dalley et al., 2011), strongly interacting with the ACC (Mechelmans et al., 2017), and implicated in response inhibition and motor control (Morris et al., 2016; Neufang et al., 2016; Mechelmans et al., 2017). Association between *H* and the frontal cortex have been reported in numerous human studies before. For example, *H* in the prefrontal cortex in healthy volunteers correlated with impulsivity (Gentili et al., 2020), personality traits (e.g., extraversion Lei et al., 2013; Gentili et al., 2017), cognitive processing (response time in a face recognition task Wink et al., 2008), and healthy aging (Dong et al., 2018; Mukli et al., 2018). In addition, pathological processes were discovered, e.g., in the IFG of schizophrenic patients (Sokunbi et al., 2014), the MFG of patients with mild cognitive impairment (Long et al., 2016, 2018), and Alzheimer’s Disease (Nimmy John et al., 2018) as well as the IFG of autistic individuals (Lai et al., 2010). Thus, frontal processing has been described as following fractal rules before. In line with our findings, *H* seemed to be able to detect neural alterations not only in pathological populations but also in individual variability in the normal population (Serletis et al., 2012).

In summary, in this study, we addressed the relation between altered *H*_*fGn*_ and further neural network parameters in an explorative way to get an idea of how deviations in *H*_*fGn*_ were associated with neural network functioning. We found that alterations in *H*_*fGn*_ were predominantly related in fronto-hippocampal pathways arguing, that *H*_*fGn*_ proved its sensitivity toward learning and memory processing. However, despite the highly interesting and plausible results, we have to state, the current findings reflect processes within a very specific sample (young healthy male students) performing the also very specialized 5-CSRTT (a paradigm which has, to date, predominantly been used in animals). In addition, in contrast to earlier publications, where the interaction between several physiological systems such as brain and cardiac system (Liu et al., 2015, Lin et al., 2016), or brain, cardiac and respiratory systems (Bartsch and Ivanov, 2014) were addressed over phases of different physiological states, analysis of the current work was limited to one physiological network and only during one single state. Especially the frontal lobe seemed to interact with the heart as has been shown in several studies by Thayer et al. (2009, 2012) and Patron et al. (2019). In addition, this data stems from a sample of male subjects only. The data has been collected in a pilot study, which has been published 2016 as the first fMRI study using the human version of the originally animal paradigm 5-CSRTT (Neufang et al., 2016). As at that time, network regions associated with WI in humans was mainly theoretical, we decided to investigate neural underpinnings in a highly homogenous sample, which is healthy male students. Thus, findings are of limited generalizability and need to be replicated in future studies with experimental protocols like those published before. However, the combination of *H* with further cognitive, neural and peripheral parameters such as inflammation scores as well as the longitudinal study of *H* to describe physiological variations (e.g., diurnal, brain maturation, aging) are of highest interest in the study of neural networks.

## Data Availability Statement

The datasets generated for this study are available on request to the corresponding author.

## Ethics Statement

The studies involving human participants were reviewed and approved by Ethics Committee of the Faculty of Medicine, University of Würzburg. The patients/participants provided their written informed consent to participate in this study.

## Author Contributions

AA was responsible for *H* data analysis. SN performed brain activation and connectivity analyses. All authors equally contributed to manuscript writing.

## Conflict of Interest

The authors declare that the research was conducted in the absence of any commercial or financial relationships that could be construed as a potential conflict of interest.

## Footnotes

1 http://marsbar.sourceforge.net/marsbar.pdf

2 http://fmri.wfubmc.edu/software/pickatlas

## Supplementary Material

FIGURE S1 A representative experimental trial.

TABLE S1 DCM families and models.
