# Supplementary figures and images for: Regional Hurst Exponent Reflects Impulsivity-Related Alterations in Fronto-Hippocampal Pathways Within the Waiting Impulsivity Network
Source: Front Physiol. 2020 Jul 10;11:827. doi: 10.3389/fphys.2020.00827 (PMC7381286; doi:10.3389/fphys.2020.00827)

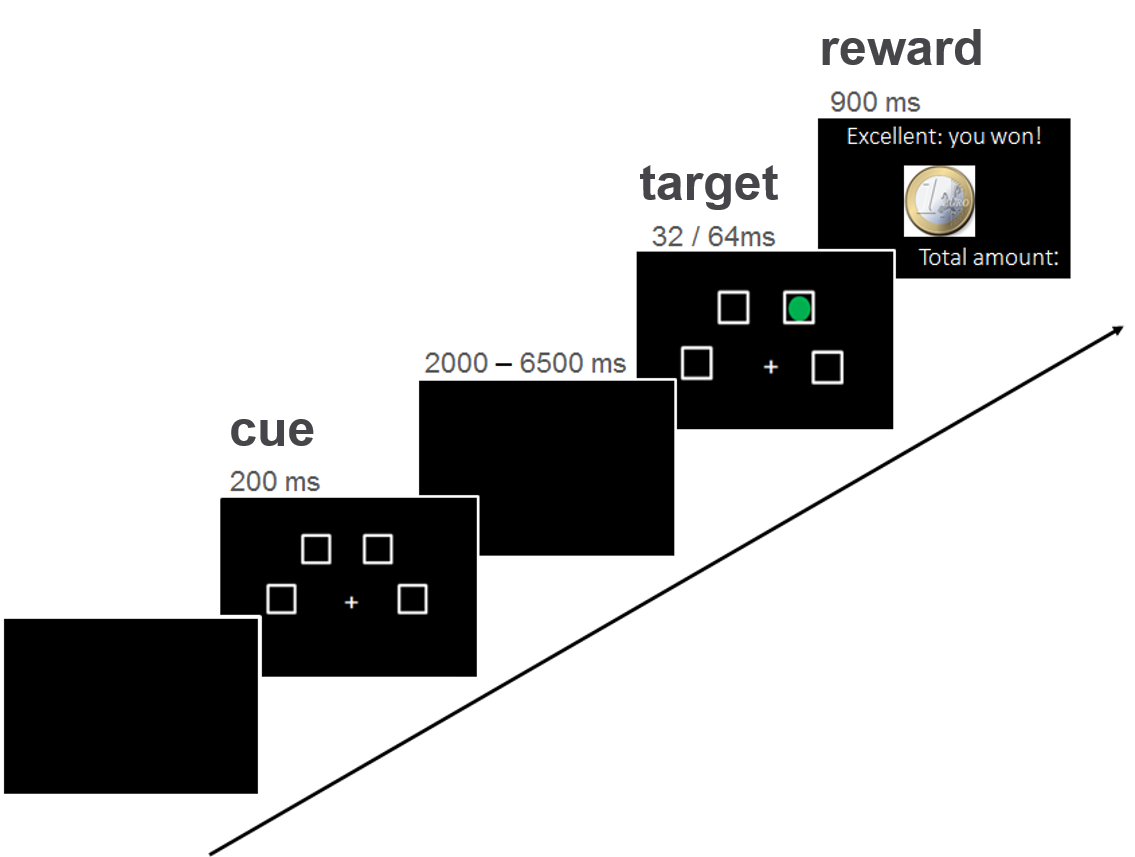

Supplement: FIGURE S1 — A representative experimental trial. [file Image_1.TIF]
