# Supplementary material for: Regional Hurst Exponent Reflects Impulsivity-Related Alterations in Fronto-Hippocampal Pathways Within the Waiting Impulsivity Network
Source: Front Physiol. 2020 Jul 10;11:827. doi: 10.3389/fphys.2020.00827 (PMC7381286; doi:10.3389/fphys.2020.00827)
Supplement: TABLE S1 — DCM families and models. [file Table_1.DOCX]

**Table s1.** DCM families and models

| **condition** | **# family** | **# model** | **Family name** | **Model name** |
| --- | --- | --- | --- | --- |
| response inhibition | 1 | 1 | HC bottom-up | bilat HC🡺MFG |
|  |  | 2 |  | bilat HC🡺ACC |
|  |  | 3 |  | bilat HC🡺MFG+ACC |
|  | 2 | 5 | MFG top-down | bilat MFG🡺r/l_HC |
|  | 3 | 6 | ACC top-down | ACC🡺r/l_HC |
|  | 4 | 7 | MFG+ACC top-down | bilat MFG+ACC🡺r/l_HC |
| reward | 5 | 8 | NAcc bottom-up | NAcc🡺vmPFC |
|  |  | 9 |  | NAcc🡺l_AMY |
|  |  | 10 |  | NAcc🡺l_AMY+vmPFC |
|  | 6 | 11 | l_AMY bottom-up | l_AMY🡺vmPFC |
|  |  | 12 |  | l_AMY🡺NAcc |
|  |  | 13 |  | l_AMY🡺NAcc+vmPFC |
|  | 7 | 14 | NAcc+l_AMY bottom-up | l_AMY+NAcc🡺vmPFC |
|  | 8 | 15 | vmPFC top-down | vmPFC🡺NAcc |
|  |  | 16 |  | vmPFC🡺l_AMY |
|  |  | 17 |  | vmPFC🡺NAcc+l_AMY |
| across conditions | 9 | 18 | NAcc bottom-up | NAcc🡺MFG |
|  |  | 19 |  | NAcc🡺MFG+ACC |
|  |  | 20 |  | NAcc🡺MFG+HC |
|  |  | 21 |  | NAcc🡺MFG+HC+ACC |
|  | 10 | 22 | l_AMY+NAcc bottom-up | l_AMY+NAcc🡺MFG |
|  | 11 | 23 | MFG top-down | bilat MFG🡺NAcc |
|  |  | 24 |  | bilat MFG🡺 NAcc+l_AMY |
|  | 12 | 25 | ACC top-down | ACC🡺NAcc |
|  |  | 26 |  | ACC🡺 NAcc+l_AMY |
|  | 13 | 27 | MFG+ACC top-down | bilat MFG+ACC🡺NAcc |
|  |  | 28 |  | bilat MFG+ACC🡺 NAcc+l_AMY |
